# Supplementary material for: Mobile primary healthcare for post-COVID patients in rural areas: a proof-of-concept study
Source: Infection. 2022 Jul 13;51(2):337–45. doi: 10.1007/s15010-022-01881-0 (PMC9281342; doi:10.1007/s15010-022-01881-0)

Supplemental Figure 1: Subgroup analysis of SF36 based on patient sex

Supplemental Figure 2: Subgroup analysis of SF36 based on patient age


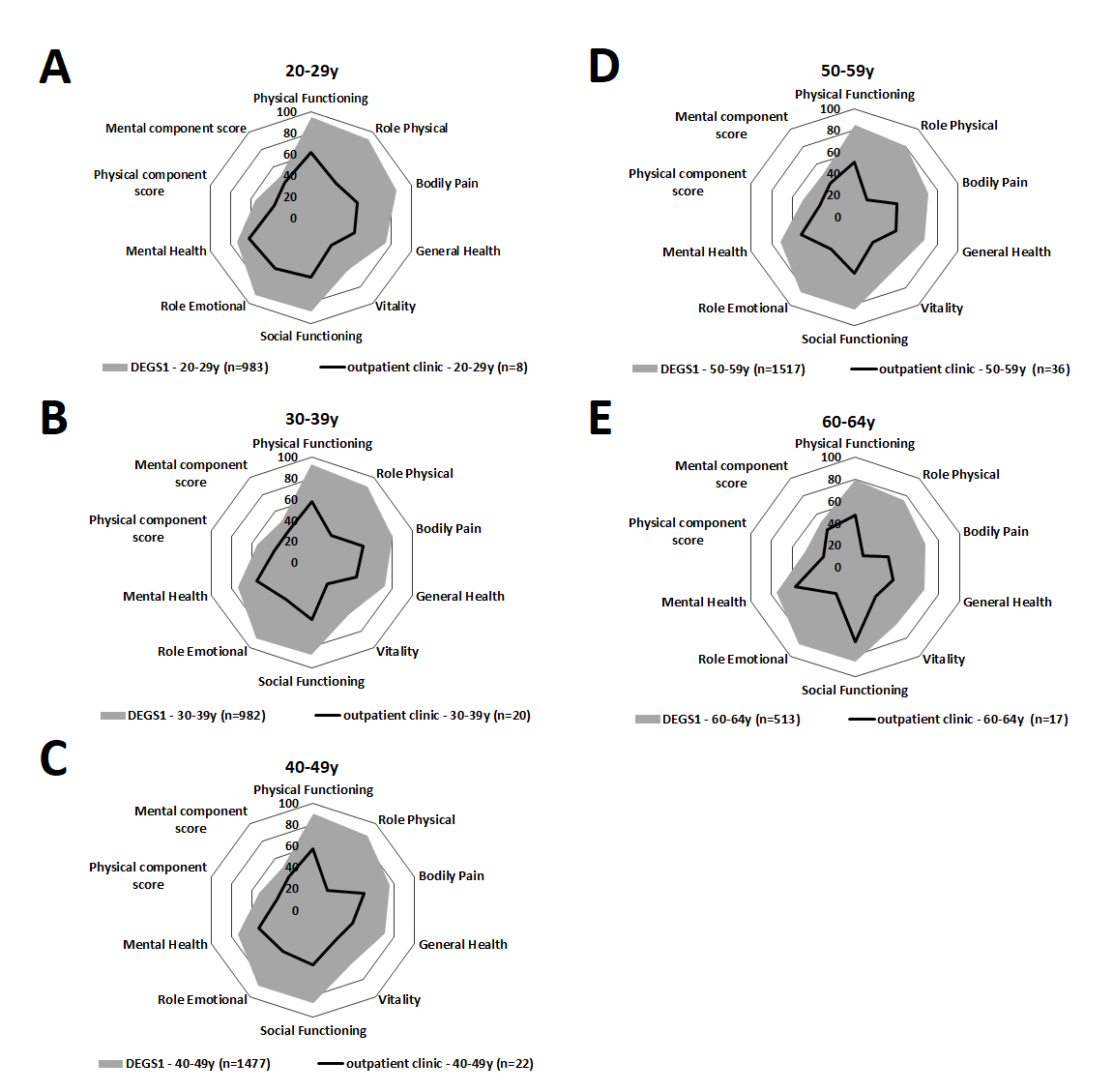

Supplement: Supplementary file 1 — Supplementary file1 (DOCX 178 kb) [file 15010_2022_1881_MOESM1_ESM.docx]
